# Lifetime and world-time in accelerated medicine. An essay on the consolation of synchronisation

**DOI:** 10.1007/s11019-025-10309-z

**Published:** 2025-11-21

**Authors:** Urban Wiesing

**Affiliations:** Institute of Ethics and History of Medicine, University of Tübingen, Tübingen, Germany

**Keywords:** Acceleration, Progress, Lifetime, World-time, Synchronization, Consolation

## Abstract

The essay examines how prominent scientists, politicians, and managers respond to the condition of human existence that a human being is only a short time in the long time of the world. In connection with the accelerated medicine and its predicted progress, they segment the future and imagine themselves at a new stage of humanity. They strive for a. privileged relationship to the world by claiming to conclude humanity’s prehistory in the foreseeable future, if possible, in their own lifetime. They are trying to synchronise their lifetime with the world-time. In this way they hope for consolation, draw on old religious motifs and attribute religious elements to medicine.

## Introduction

Ars longa, vita brevis. Life is short, and the Art long (Corpus Hippocraticum, Adams 1886, p. 192). Even the first Hippocratic aphorism, around 2500 years old, points to a fundamental constellation of human existence: One’s own life is short compared with the skill developed by humans, that is, the medical craft. One only lives for a certain time, the ‘medical arts’, however, will continue beyond one’s own life. As a doctor one can only learn and practice a mere portion of the perennial medical arts, for the duration of a short life. This difference between a lifetime and the possibilities of the medical arts is increasing in modernity and especially in view of the acceleration of science. The arts grow much faster than the average lifespan is extended. In turn, a smaller and smaller proportion of medical knowledge and skills can be incorporated into a career in a human life.

In the early 20th century, Max Weber took up this constellation for the sciences in general, asking about the meaning of a life as a researcher: “Why does one engage in something that, in actual fact, never comes to an end, and never can?” (Weber 1985, p 487). For researchers are ever part of a process that has no end, contrary to one’s own life that will assuredly cease. As a researcher, one works in the academic disciplines and sciences, may make one or another discovery for a limited sub-discipline or area, one that is likely to be outdated soon, retires, dies, and sciences justcontinue. One’s own attainments age agonizingly quickly in the face of rapid progress in research. At most, the best researchers are granted the opportunity to stand at the spearhead of the sciences for a short time. And then even they are inevitably surpassed by younger researchers. At best, they remain as a footnote to history.

How can one find meaning and satisfaction in such a process? Max Weber’s answer is sobering: It is the purpose of scientific work to constantly evolve and encounter new questions. It “*demands* to be surpassed and rendered obsolete. Everyone who wishes to serve science will have to resign himself to this” (Weber 1985, p. 487, emphasis orig.). So, nothing remains but to “resign” oneself! This sobering answer would be hard to bear for many researchers of modern medicine.

A similar constellation has been examined by Hans Blumenberg who differentiated between the own life-time and the time of the world (Blumenberg 1986). A human knows that time was before him and after him. The world did not begin with this person and will not end with them. Furthermore, the world is silent and indifferent about this fact. When a person dies, the world simply continues to revolve. As such, a human exists only for a short time span, his lifetime (*Lebenszeit*), lost in a moreover uninfluenceable world-time (*Weltzeit*). Or, as Thomas Nagel puts it, “our lives are mere instants even on a geological vastness of the universe, let alone a cosmic one” (Nagel 1971, p. 717). Most humans will be soon forgotten after their deaths, the world will continue to exist without them. This is a cause of enormous offense to them.

Humans have ideas and wishes that go far beyond their lifetime. The bifurcation between lifetime and the possibilities of world-time diverges even further under the conditions of acceleration. “Less and less time for more and more possibilities and desires” (Blumenberg 1986, p. 73, own translation). What can one do to mitigate this? Humans try to compensate by works in their lifetime that have an effect beyond their lifetime and so make an impact on world time. And they want to synchronise the end of their lives with a new stage of humanity. They hope that this will give them consolation.

Hans Blumenberg also differentiates between ‘must-do’ time (*Musszeit*) and ‘can-do’ time (*Kannzeit*). Must-do time is necessary for self-preservation, such as procurement of food, protection from cold, rain, enemies etc. Can-do time, on the other hand, constitutes a ‘time-margin of indefinite fulfillments’ (Blumenberg 1986, p. 291). People in modern industrial society must invest less time in their self- preservation. Their progress shows in the proportional shift of must-do time towards can-do time (ibid.).

So: Life is short, the Arts are long, the arts and sciences grow more quickly in proportion to the lifetime, the divergence between a lifetime and the possibilities that the world offers is increasing. How does a researcher in these accelerated sciences deal with this? In this essay some contemporary answers regarding the increasing discrepancy between lifetime and time of medicine are investigated. The aim here is not to provide a complete overview of all answers given today to this constellation. There are numerous other attempts to deal with the finiteness of one’s own life than those examined here. But the ones presented here, these striking responses of some important protagonists of current technological developments are interesting because of their structures and recurring religious motifs from past times. What do some contemporary scientists, managers, and politicians do to mitigate the increasing divergence of lifetime and world-time? What do they do when digitalization, artificial intelligence, molecular medicine, genetics, and their convergence enhance the speed of knowledge acquisition and are meant to change medicine quickly and fundamentally? Four reactions to this constellation are presented here:

1. Scientists, managers, and politicians segment the future. In this way, they create the precondition for synchronizing their lifetime with world-time for important medical achievements at least. It is their aspiration to create and witness a new segment of world-time, a new stage of humanity, during their lifetime.
2. Thereby, acceleration becomes ambivalent: It lets a scientist become obsolete even faster but compensates by the prospect of the synchronization of lifetime with a new stage of world-time.
3. This leads to the fact that can-do time becomes must-do time again. One must always work to reach a new threshold of world-time faster and to still experience it.

In this way, these scientists copy early Christian conceptions of the last judgment that is to come within the lifetime. Medicine promises salvation even during lifetime; it takes on substitutive functions of religion. Medicine thus becomes a science of salvation. These responses regarding the characteristics of an accelerated medicine are explained in the following sections.

## Segmenting the future

The convergence of digitalization, AI, molecular medicine, and genetics leads to euphoric ideas of the future of medicine and several promises about what medicine will invent next. These assurances segment the future by promising new medical innovations which promise a new step for humankind.

Some well-known examples may illustrate this: For instance, Mark Zuckerberg is “optimistic that it is possible to cure, prevent or manage all disease in our children’s lifetime” (Zuckerberg 2016). So, they will supposedly have left the prehistory of humankind with its suffering due to disease behind them. Zuckerberg’s children will live with an entirely new level of health.

Aubrey de Grey, a famous gerontologist, is firmly convinced that ageing can be abolished within the next 30 years. People will then live an average of 1000 years – at least! When asked if he would live to see it, de Grey replied: “I am doing my best, and the chances are not bad at all. The prospect of suppressing ageing is only 30 years away” (see Stirn 2025, p. 3, own translation).

Ray Kurzweil, chief engineer at Google among other things, predicts a state of singularity for 2045. Then artificial intelligence will supposedly have overtaken human intelligence. What exactly will happen then is unknown. But humans would likely be immortal since they could then be represented completely on a computer. Kurzweil, born in 1948, leads a highly prevention-oriented lifestyle taking numerous pills in the hope that he will live to see it.

So, Zuckerberg, de Grey, and Kurzweil are trying to synchronize a new level of world-time with their own lifetime by contributing to progress while concomitantly segmenting the future. They hope that they or their children will live in a new era of humankind.

Certain projects making similar promises ought to be mentioned besides certain people. A particularly striking example of concrete promises of segmentation in the future is the ‘war on cancer’, which was promoted by the Lasker Foundation in major advertising campaigns (The Mary Lasker Papers 2019). Hopes and convictions that cancer could be defeated soon had already existed frequently before, just think of the example of William F. Koch and his ultra-dilute “synthetic antitoxins” in the first half of the 20th century. (Young and MacFadyen 1998) But in 1969 an explicit ‘war on cancer’ began and was supposed to be won by the 200th birthday of the USA, i.e. in 1976. By then, cancer was supposed to be extinct. After that did not work, 2015 was given as the next cut-off point for cancer suffering to have disappeared forever, promised by the top American oncologist, Andrew C. Eschenbach before the US Senate in 2005. Or in 2012, if the US Senate would only approve additional money (Congressional Committee Materials 2006). As if that were not enough, Israeli scientists promised in January 2019 that they would be able to treat cancer “completely” within one year, i.e. 2020 (Jaffe-Hoffman 2019). So far, that has not materialized. Microsoft has committed itself to 2026 for defeating cancer (Knapton 2016). Manfred Weber, the people’s parties’ top candidate for the European Parliament, predicted another 5 to 10 years in 2019 (Weber 1985, p. 5). Likewise in 2019, the German Federal Minister of Health, Jens Spahn, assumed that cancer would be defeated in 10 to 20 years (Bröcker and Quadbeck 2019).

What do they all have in common? Fixed points in time are given regarding when cancer will be defeated completely: 1976, or 2012, or 2015, at least 2020, at the latest 2026, at the very latest 2029, at the very-very latest 2039. Then a new stage of humanity will have been reached. The prehistory of humanity encompassing deaths by cancer will be over. And one might still experience the crossing of this threshold, perhaps even be involved in it, as a researcher, entrepreneur, politician. One’s own efforts would lead to one’s own lifetime coinciding with a threshold of humanity. But the reality in the war on cancer is different and many assessments that a measure would defeat cancer have not yet materialised: “In fact, there have been more than 200 failures of supposed cancer breakthroughs in recent years” (Belluz 2019). Certainly, there are new therapies, including effective ones and new therapeutic approaches, but the number of people dying from cancer continues to rise on a global scale. For 2018 alone, Global Cancer Statistics (Bray et al. 2018) calculated at least 9.5 million cancer deaths worldwide, with numbers continuing to rise.

Absurdly precise forecasts are made in these proclamations of a new stage in human history. The future is segmented by specifying exact times – by no means only in the ‘war on cancer’: At the beginning of the Human Genome Project, it was promised that medicine would be genetic medicine by the year 2015 and thus much more successful than ever (Collins 1999). However, this has not come to pass. In 2015, the German daily newspaper *Die Welt* reported that there would be no congenital diseases anymore in 2050: “Geneticists are sure of that” (Lossau 2015, own translation). Knowing how dynamic and complex scientific progress is, there is no way of being certain 35 years beforehand that congenital diseases will disappear. Likewise, it was proclaimed with the same precision: The fully automated robot-surgeon will revolutionize medicine exactly in the year 2053. How would any “leading AI-researcher” (Schulz 2018, p. 85) be able to know that this would be available in 2053 rather than in 2052 or 2054? How is it even possible to calculate that so precisely 35 years in advance?

2053 seems to be a distinct year for such announcements. W. French Anderson, one of the pioneers of gene therapy assumes: “By 2053, there will be a gene-based treatment for essentially every disease. Cancer, heart disease, and other modern-day scourges will be vastly reduced” (in McCain 2005, p. 52). Here, too, the question is: How can one know so precisely, about 50 years in advance, that a new stage of humanity will have been reached? And, as mentioned, similar things were promised for 2015 when the Human Genome Project was launched. All these examples have in common that they not only declare a new stage of humanity but also predict to the exact year when it will occur. But why are the new stages of humanity predicted so precisely for a specific year? Precisely dated predictions are more convincing, redemption becomes foreseeable, one can anticipate it with calendarial exactitude.

To conclude this point, an interesting variation regarding the synchronization of world-time and lifetime ought to be mentioned, that of Steve Jobs. When he fell ill with pancreatic cancer, in line with his technological optimism he underwent genetic examinations and hoped that a curative therapy could be derived from genome sequencing, which was still very complex and expensive at the time. He placed the greatest therapeutic hopes in this diagnostic technology. He was convinced that he would either be one of the first to survive cancer through this technology or one of the last to die from cancer (see Schulz 2018, p. 124). In any case he linked his lifetime with world-time: Either he would be one of the last victims of mankind’s medical prehistory in which people still die of cancer. Or he would be one of the first to benefit from the concluded medical prehistory. Either he would be one of the last unfortunates or one of the first redeemed. And even if not in an obvious medical sense, he has contributed significantly to the technological leap into a new era through his entrepreneurial activities. He was able to shape history (see Schulz 2018, p. 124). Either way, he segmented time and positioned himself at this threshold. Steve Jobs died of cancer in 2011 but likely not as one of the last ones. To summarize the initial reaction to the gap between world-time and lifetime: Some leading representatives of a new type of medicine, as well as entrepreneurs and politicians, try to synchronize world-time with their own lifetime by segmenting the future. They do this by promising new, world-changing achievements during their lifetime (or the lifetime of their children). Realistically, it is part of the business of applying for research funding and attracting investment that too much is promised. But this is a special form of overpromising: Such individuals strive for a privileged relationship to the world by claiming to conclude humanity’s prehistory in the foreseeable future, if possible, in their own lifetime. They see themselves as the last generation of human prehistory. In Silicon Valley and other places of accelerated science, meaning in life is tied to changes in world-time. People there are successful, often have good jobs and a comfortable livelihood. But what lies beyond that? What remains for eternity? The ostentatious denial of meaning in modernity is countered by a sense of meaning that comes from being involved in and experiencing breakthroughs in world-time.

Presumably, the protagonists will also gain financially from their endeavours if they are successful. Nevertheless, in their statements this aspect is not the main motivation. They are primarily concerned with the meaning of their lives. Their strategy for dealing with human mortality is interesting: the aforementioned protagonists of the new technical developments do not address the question of what mortality could mean for humans, including in terms of benefits (see e.g. Kaas 1985; Hägglund 2019). They estimate the mortality of human beings negative and rather want to mitigate the (still) existing mortality by providing consolatory meaning for people by synchronization of world-time with their own lifetime. Moreover, they continue to assume that for a certain time humans are still mortal, because if men were immortal the consolation offered would be pointless because they do not die.

## Acceleration is becoming ambivalent

Unfortunately, successful synchronization of lifetime and world-time is not certain. The development of these redeeming technologies might possibly take longer than one’s own lifetime; as shown by the example of Steve Jobs. Missing synchronization can be reduced by two strategies. Both strategies can be found in the stories about the future of new medicine:

1 st strategy: Prolonging one’s own life; this increases the probability of still experiencing a new stage of science.

As mentioned before: Ray Kurzweil takes numerous pills and leads a highly prevention-oriented lifestyle in the hope of living longer and experiencing the historic leap. And many follow him in this lifestyle. Next to it many start-ups in Silicon Valley focus on longevity and a lot of money is being invested in this strategy. Longevity and the conviction that it can be achieved in the foreseeable future through new technologies and a corresponding change in lifestyle became an “obsession” (Ward 2022) in Silicon Valley.

One group however is less affected by this: young scientists. They have a higher chance of synchronisation with the threshold of world history they predicted. A typical example is the ‘child prodigy’ Sam Ginn. While still a student, he already owned a start-up company at the age of 20, as is par for the course in Silicon Valley; he works on artificial intelligence and is firmly convinced: “This kind of Deep Learning will one day undoubtedly be considered a revolution: There is a time before and a time after.” (Scheu, 2019, own translation) At the age of 20, such ‘high flyers’ can certainly expect to enjoy the “time after” in their own lifetime. For the older ones, however, the outlook is not as good. They must endeavour to prolong their lives.

2nd strategy: Working on the quickest possible advancement of a new threshold in world-time. Accelerate the acceleration!

It must be kept in mind that the sciences are already in a phase (as is technological and social development) that is characterised by acceleration. (Rosa 2010). More and more knowledge is being produced in less and less time. Consequently, existing knowledge is becoming obsolete faster than ever. specialisation is also progressing. We know more and more about smaller and smaller areas. This acceleration, however, is ambivalent as it has also negative and paradoxical side effects. People are living in a state of perpetual anticipation (Virillio 2006). Quickly changeable and less changeable elements collide. Because people cannot always change as quickly as science or the social environment does, “de-synchronisations” can occur (Rosa 2010, p 69). One’s own ability to change is not always commensurate with change in the sciences. This in turn means that scientists are condemned to lifelong learning anyway and nevertheless cannot prevent being overtaken by younger colleagues in terms of expertise earlier in professional life and. Acceleration increases and of alienation from one’s own work during the professional life.

But regardless of this: If one wants to experience a threshold of scientific progress, a further acceleration of the sciences can increase the likelihood of this happening. Therefore, these people should work on accelerating progress that is already rapid. This reinforces the ambivalences: The already existing pressure for acceleration with its known risks and side-effects is increased even further. There is the danger of becoming obsolete even quicker as a researcher because of the accelerated technological developments; one’s own accomplishments will be even more quickly outdone. But a prize awaits: one might experience a new step in world-time. Acceleration therefore “serves as a strategy to erase the difference between the time of the world and the time of our life“ (Rosa 2010, p. 30). However, an increased probability that one’s own life is synchronized with a new era in the history of mankind is only available at the price of further acceleration.

## Can-do time becomes must-do time again

In Silicon Valley, the prototype, but also in numerous other places of accelerated science, a special work ethic prevails. People work all the time, are satisfied with that and display as much. One would not have to work as much as is usual in the Valley to earn a living. What is behind that? Where does this attitude about work come from? When having such far-reaching goals as in the accelerated sciences, then one must combine all forces to bring these about. Because one stands on the verge of a decisive step in human history, can-do time becomes a new must-do time. The goals are so high that one *must* constantly strive towards them. High-ranking goals for humanity are so imminent that one must always work towards this attainment. Whoever does not labour constantly could not be among the first to bring forth salvation. This is exactly what is happening in Silicon Valley, where these protagonists at least try to “force world-time to conform to the measure of lifetime, to fix the limit of their life on the moment that a certain completion for the world is conceded” (Blumenberg 1986, p. 73, own translation). The protagonists see themselves as accelerators of the coming redemption. Therefore, the tenet is: Do not wait, but act, and do so always. The expectation of end-times accelerates and intensifies action.

The fact that can-do time is once again becoming must-do time is paradoxical insofar as modernity also is characterized by the increase of the “time margin of indeterminate fulfillments” (Blumenberg 1986, p. 291, own translation) compared to must-do time. Technology is supposed to relieve people of must-do time and grant more can-do time. But the developers of such technologies have to work constantly, otherwise the new threshold of human history might come later, and the synchronization of lifetime and the new medical world-time would be missed.

## The protagonists of medical progress copy early Christian ideas of the last judgement

The examples presented above of how protagonists of modern medicine want to console themselves by securing a special place in history, namely on the threshold of a new stage of humanity, all relate to the present and are linked to technological progress. However, the basic motif of dividing time into epochs and linking lifetimes with a new stage of humanity is by no means new. Structuring the future into a prehistory and an approaching phase of redemption shows a clear similarity to Christian eschatology, to the doctrine of the completion of creation. A characteristic change from antiquity by early Christianity is the relation to time (see Benz 1977). Unlike ancient religions in which time simply flowed, Christianity segmented time. Accordingly, the history of mankind is divided into three different periods: first the time before the birth of Christ, then the time from the birth of the Saviour until the Last Judgement which was to come soon, and the time after. There was still much to be done before the Last Day, the return of Christ who would come soon, therefore haste was required.

The parallels to visions of the future in contemporary medicine are obvious; they too are characterized by the accelerating effect of end-time expectation (see Benz 1977, p. 16). First, the event takes place that has a drastic impact on the course of history: Genetic engineering, especially CRISPR/Cas, Artificial Intelligence and other technologies are discovered. They set out to change the world. Thereby a new age has dawned that distinguishes itself from the previous ones in that we supposedly know and can do something of utmost importance. The final act of humanity’s prehistory has begun. Or, as Harari puts it in his *History of Tomorrow*: “The Last Days of Death” (Harari 2017, p. 21) have commenced!

Admittedly, there are differences: Judgment Day in the Christian religion comes solely by God’s decree, and it is best to be well prepared for it. In contrast, the salvation of new medicine is the result of scientific progress, that is, human activity. “We don’t have to wait for the Second Coming in order to overcome death” (ibid., p. 23) Which, on the one hand, only leads to changes in human beings in this world (Hoffmeister 2019, p. 61) and not in the hereafter, and, on the other hand, is dependent on collective human efforts. The insightful have known this for a long time and that is why they are labouring with the utmost vigour. Can-do time becomes must-do time, for the sake of redemption.

The early Christians conceived themselves as having the hope of a certain synchronicity of their lifetime and world-time, being in a state of imminent expectation. They believed themselves to be in a privileged relationship to the world. The return of Christ on the Last Day, *parousia*, was supposed to take place during their lifetime. This hope has as yet not been fulfilled. Nonetheless, it can be observed again in the narratives about the future by contemporary medicine. The dimension of the promised changes is, to say the least, similar: Through science and technology, one seeks a privileged relationship to world-time. Both the Christian religion and modern medicine promise a new, completely different era that will soon come. Judgment Day is imminent or – according to the technological, modern variant – a new stage of earthly human history, for instance, freedom from cancer, the disappearance of all genetic diseases, indeed of all diseases, or overcoming a meagre life expectancy of about 80 years on average, perhaps even death.

These ideas are also to be interpreted as stories of how one deals with the characteristics of one’s own life and makes a story out of it, a comforting, a redeeming one. They are stories with the claim to coherence, creating a structure of past, present, and future, which can also be understood as stories of meaning. People give meaning to their existence through a certain narrative construction in the face of the pressing need for meaning (see Ricouer 1988).

These narratives, as told in a typical narrative by individuals involved in the technological progress of medicine, is interestingly intertwined with broader theological and philosophical concerns about finality, death, consolation and salvation. Here we find unexpected analogies of contemporary technical and medical ideas for the future and early Christian ideas. But these narratives of giving meaning to one’s own life use medicine for a task that it cannot fulfil due to its constitution. The ideas of a synchronized future show once again that modern medicine has long since been assigned functions that far exceed the responsibilities and capabilities of science. With medicine, one tries to mitigate the affront from the difference between world-time and lifetime. The pronounced denial of meaning in modernity is countered by a sense of meaning that is oriented towards involvement in or proximity to breakthroughs in world- time, towards synchronization (Wiesing 2019). As said: Ars longa, vita brevis. But if a short life coincides with a time when the ‘long art’ makes a new threshold of humanity possible, and one is also involved in it, then that at least is comforting. Medicine is assigned to the task of redemption, becoming a science of salvation. But then it takes on a function which is in contradiction to their approach and methods. That medicine is nevertheless attributed to this role may also be because there are fewer institutions that can credibly promise salvation in this modern age.
